# CLRS: Continual Learning Benchmark for Remote Sensing Image Scene Classification

**DOI:** 10.3390/s20041226

**Published:** 2020-02-24

**Authors:** Haifeng Li, Hao Jiang, Xin Gu, Jian Peng, Wenbo Li, Liang Hong, Chao Tao

**Affiliations:** 1 School of Geosciences and Info-Physics, Central South University, Changsha 410083, China; lihaifeng@csu.edu.cn (H.L.); jh001100@csu.edu.cn (H.J.); PengJ2017@csu.edu.cn (J.P.); 2 China Academy of Launch Vehicle Technology Research and Development Center, Beijing 100076, China; nync396@126.com; 3 Institute of Technology Innovation, Hefei Institutes of Physical Science, Chinese Academy of Sciences, Hefei 230088, China; wbli@iim.ac.cn; 4 School of Tourism and Geography, Yunnan Normal University, Kunming 650500, China; hongliang@ynnu.edu.cn

**Keywords:** scene classification, continual learning, remote sensing dataset, CLRS

## Abstract

Remote sensing image scene classification has a high application value in the agricultural, military, as well as other fields. A large amount of remote sensing data is obtained every day. After learning the new batch data, scene classification algorithms based on deep learning face the problem of catastrophic forgetting, that is, they cannot maintain the performance of the old batch data. Therefore, it has become more and more important to ensure that the scene classification model has the ability of continual learning, that is, to learn new batch data without forgetting the performance of the old batch data. However, the existing remote sensing image scene classification datasets all use static benchmarks and lack the standard to divide the datasets into a number of sequential learning training batches, which largely limits the development of continual learning in remote sensing image scene classification. First, this study gives the criteria for training batches that have been partitioned into three continual learning scenarios, and proposes a large-scale remote sensing image scene classification database called the Continual Learning Benchmark for Remote Sensing (CLRS). The goal of CLRS is to help develop state-of-the-art continual learning algorithms in the field of remote sensing image scene classification. In addition, in this paper, a new method of constructing a large-scale remote sensing image classification database based on the target detection pretrained model is proposed, which can effectively reduce manual annotations. Finally, several mainstream continual learning methods are tested and analyzed under three continual learning scenarios, and the results can be used as a baseline for future work.

## 1. Introduction

With the rapid development of remote sensing imaging technology, high-resolution remote sensing images are easily obtained and the interpretation of remote sensing images has evolved from the pixel- to the scene-level [1,2]. In this study, we focus on the problem of remote sensing image scene classification based on deep learning, which has been widely used in urban planning, disaster monitoring, and other fields, to provide a high-level interpretation ability for high-resolution remote sensing images. It has become an important research topic and has received extensive attention from researchers [3,4,5,6,7].

The existing classification algorithms, based on deep learning, assume that all of the training data were available before the training began. This is not realistic in real world remote sensing image scene classification, as a large number of new remote sensing images are obtained from different satellites every day. Researchers have done a good deal of work on this problem. For example, Haikel [8] proposed a framework for multitask learning that can learn from all available datasets. However, this approach requires joint training using multiple datasets and the storage of historical data, resulting in wasted storage and computing resources. Liu et al. [9] used online algorithms to incrementally process constantly updated micro-video data, effectively improving the real-time performance of the algorithm. However, the existing scene classification algorithms still have a serious drawback that, once the model has been trained on the new data, there will be a catastrophic forgetting problem [10,11,12]. That is, the model cannot maintain its performance of previously learned data because the parameter space of the old data is overwritten. Therefore, it is a challenge for remote sensing community to keep the performance of old data while learning new data.

Recently, continual learning [13] has made a great contribution to solving the above two shortcomings. Continual learning aims to continuously learn new tasks without forgetting the ability to perform previously trained tasks, and does not need to store the historical data of old tasks, thus greatly saving storage space. In recent years, researchers have proposed a number of emerging approaches to continual learning. In the field of computer vision, James et al. [14] proposed a regularized strategy to avoid catastrophic forgetting by penalizing changes in parameters that were important to the old task, while learning the new one. The same work has been extended in Reference [15]. The researchers proposed an online method for calculating the importance of parameters, which saves memory space by not saving the importance matrix. Li et al. [16] proposed a method of knowledge distillation that reduces catastrophic forgetting by continuously reinforcing the stability of model output on old tasks, etc. In the field of remote sensing, only Tasar et al. [17] applied the continual learning method to the semantic segmentation of remote sensing images. Remote sensing scene classification working with continual learning is a topic that has not been explored yet. Therefore, it is very urgent and important to ensure deep learning models have the ability for continual learning, to be able to solve the disadvantages of remote sensing image scene classification algorithms in practical applications.

However, there is one major issue that seriously limits the development of continual learning in remote sensing image scene classification. There are no proper datasets and benchmarks in remote sensing to evaluate and compare emerging continual learning methods. It is important for continual learning to split the dataset into several sequential training batches according to certain criteria. However, existing scene classification datasets, such as UC-Merced [18], AID [19], NWPU-RESISC45 [20], etc., are only divided into two parts: A separate training set is used for model training and a separate test set is used for performance evaluation. These datasets are a type of “static” benchmark. A common practice in current research that uses such datasets to divide sequential training batches is to randomly divide a large dataset into several subdatasets. For example, Friedemann et al. [15] split the full MNIST [21] training dataset into five batches, each of which contains two classes. However, different researchers have different ways of dividing training batches and uniform standards for training batch partitioning are lacking, making it difficult to compare the performances of different algorithms fairly. What’s more, the application scenarios of continual learning in the real world remote sensing image scene classification are very complex. The model will encounter new classes or different instances belonging to the same class in subsequent training batches. It is clear that dealing with such complex continual learning scenarios requires specific datasets to evaluate different continual learning methods. Therefore, the existing datasets are unsuitable benchmarks for performance evaluation. In the field of remote sensing image scene classification, new datasets are urgently needed to evaluate and compare the performance of different continual learning methods.

Due to the above problem, in this paper, we proposed a continual learning benchmark for remote sensing image scene classification, called Continual Learning Benchmark for Remote Sensing (CLRS, The dataset is available at https://github.com/lehaifeng/CLRS), to solve the limitations of existing datasets. The goal of CLRS is to advance the development of the state-of-the-art continual learning algorithms in remote sensing image scene classification. To our knowledge, this is the first time that continual learning has been introduced to remote sensing scene classification. CLRS provides researchers with the label information for training batch partitioning to fairly compare and develop emerging continual learning methods, in accordance with the same criteria. In addition, we also provide a new method for constructing a large-scale remote sensing image database based on the target detection pretrained model, which can automatically detect the location information of the target scene and effectively save the manual annotation costs. Finally, the experimental results on the CLRS show that the performance of the current mainstream continual learning methods, in avoiding catastrophic forgetting, is still unsatisfactory, and thus the remote sensing community should strive to develop new continual learning methods to improve the performance of the current algorithms. In summary, the contributions of this article are summarized as follows.

(1) We analyzed three continual learning scenarios and propose training batch partitioning criteria for these three scenarios.
(2) We constructed a large-scale remote sensing image scene classification database, namely, CLRS. This database can provide researchers with better data resources to evaluate and improve the performance of continual learning methods in remote sensing image scene classification.
(3) We provided a new method for constructing a large-scale scene classification database based on the target detection pretrained model, which can save on manual annotation costs.
(4) We tested and analyzed several mainstream continual learning methods for three continual scenarios, and the results can be used as a baseline for future work.

The rest of this paper is organized as follows. We introduce the detailed construction principles and methods for the CLRS dataset in Section 2. The details of the CLRS dataset are described in Section 3. Section 4 presents the experimental results and analysis of different continual learning methods on the CLRS dataset under three scenarios. Finally, Section 5 summarizes this study.

## 2. Construction Principles and Methods of CLRS Dataset

### 2.1. Construction Principles of CLRS dataset

The application of continual learning to remote sensing image scene classification in the real world is very complicated. First, the model needs to learn multiple batches of data sequentially and cannot access data from old batches. Second, the scene classes in subsequent batches may be ones that the model has learned or ones that the model has not seen at all. We consider the classification scenarios of three continual learning scenarios that were proposed in Reference [22].

**Figure 1:**
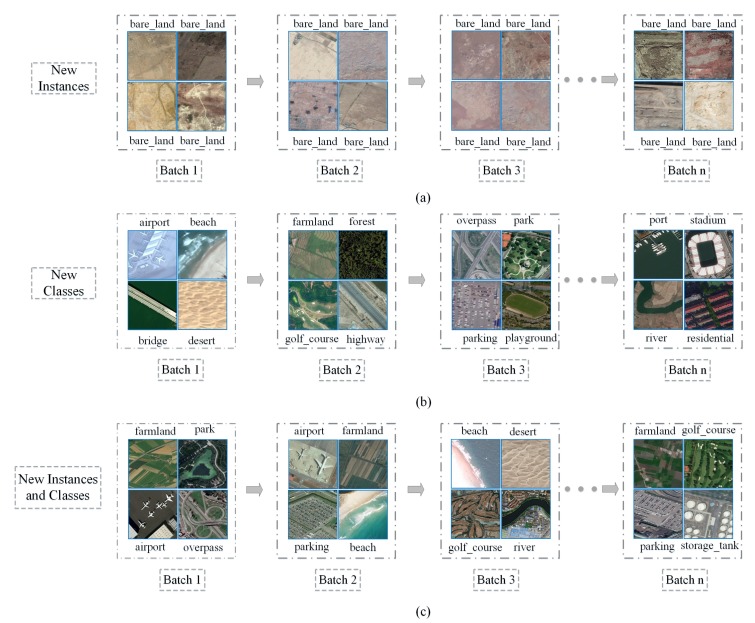
(**a**) New Instances scenario (NI): The bare_land in Batch 1, Batch 2, Batch 3, ⋯ Batch n are different in background, texture, resolution, area, etc. (**b**) New Classes scenario (NC): Different scene categories appear in subsequent training batches. (**c**) New Instances and Classes scenario (NIC): Subsequent training batches contain new scene categories and new instances of the same category.

- *New Instances scenario (NI)*: New instances of the same scene category will exist in subsequent batches. Although these instances belong to the same category, they have different textures, backgrounds, resolutions, regions, etc. (as shown in Figure 1a). In the NI scenario, the model is required to continuously consolidate the knowledge of the scene categories that have been learned to achieve better prediction accuracy.
- *New Classes scenario (NC)*: Figure 1b shows a schematic diagram of the NC scenario. The scene categories in subsequent batches are all new categories that the model has not learned before. In the NC scenario, the model must be able to quickly learn new scene categories and also not forget the knowledge of previously learned categories. In other words, the model can accurately predict new scene categories without losing the prediction accuracy for categories that have already been learned.
- *New Instances and Classes scenario (NIC)*: In the NIC scenario (as shown in Figure 1c), subsequent training batches have both new categories that the model has not learned and new instances of the classes that the model has learned. The NIC scenario is the closest to the real-world remote sensing image scene classification problem. It requires the model to correctly distinguish different scene categories and to also continuously consolidate the knowledge of the categories that have been learned. Therefore, the NIC scenario is also the most difficult of the three scenarios.

Based on these three scenarios, we propose that the construction of the CLRS should meet the following six principles.

(1) Regarding the selection of the CLRS categories, we have referenced various land-use classification standards. The authors in Reference [23] constructed a scene category network for remote sensing image scene classification (as shown in Table 1), which synthesizes various land-use classification standards, and details which subclasses are included under each parent class. From this scene category network, we select 25 common scenarios as the scene categories of the CLRS.
(2) The scene classification model based on deep learning can easily overfit small datasets. Therefore, the CLRS should have a large amount of sample data. Each class has 600 images, and the size of each CLRS image is 256×256, which will satisfy the majority of deep learning models.
(3) In practical applications of the scene classification problem, scenes in the same category will have remarkable differences due to many factors, such as illumination, background, geographical location, spatial resolution, scale, etc. Therefore, the intraclass samples of the CLRS should be diverse and representative and be able to reflect the characteristics of the scene category as truly as possible, in order to improve the robustness and generalization ability of the model. CLRS samples can be collected from multiple sensors to increase the variation of the samples within the same scene category.
(4) Many similar scenes exist in the actual scene classification. The similarity of these scenes is very high, which brings some difficulties and challenges to the classification model. Therefore, a small difference between the CLRS scene categories increases their similarity to the actual application. Similar scenarios should be considered in the selection of the CLRS categories.
(5) For the above three continual learning scenarios, the CLRS should develop a set of criteria for dividing training batches and ensure that the image data between each batch cannot be duplicated. The spatial resolution can be quantitatively divided according to its numerical value. Therefore, the CLRS should record the spatial resolution of each remote sensing image in the collection process to divide the training batches quantitatively.
(6) Remote sensing images are more complicated than natural images due to their background and texture. Therefore, the CLRS must consider the smoothness between different batches to reduce this difficulty. In other words, the training batches division should be as balanced as possible, and the differences should not be extremely large.

### 2.2. Construction Methods of CLRS Dataset

**Figure 2:**
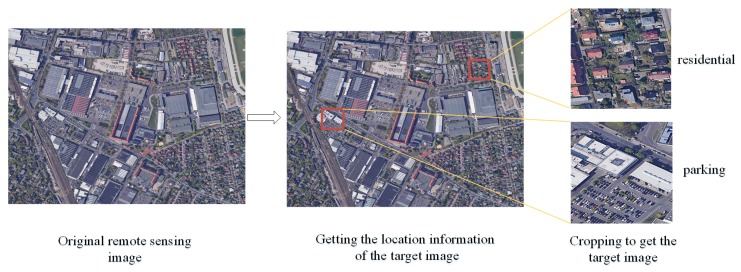
The construction process of remote sensing image scene classification database.

**Figure 3:**
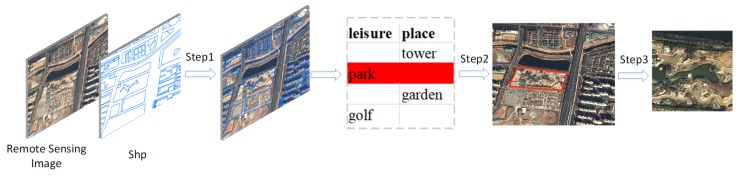
Continual Learning Benchmark for Remote Sensing (CLRS) construction process based on OpenStreetMap data. Step1: Superimposing and registering. Step2: Filtering the target area according to the OpenStreetMap (OSM) attribute. Step3: Focusing on the target area, add 10 pixels each in length and width, and crop the target image block to 256×256 size.

Obtaining the position information of target image blocks from the original remote sensing images is the key to constructing a large-scale classification database for remote sensing images. We can crop the final target image blocks based on the location information(as shown in Figure 2). The traditional methods mainly rely on manual annotation to obtain the location information of the target image blocks, which is very time-consuming. In the era of rapid development of remote sensing big data and artificial intelligence, the use of existing annotated information (such as open-source geographic attribute information data) can greatly reduce the manual annotations. In Reference [24,25], a method for constructing a remote sensing image scene classification database, based on crowdsource geographic data (OpenStreetMap data), is proposed. OpenStreetMap data are free and open-source map data can be freely annotated by anyone. The data contain attribute information annotated by people from many parts of the world. Figure 3 shows the process of constructing the CLRS based on the OpenStreetMap (OSM) data. This method can rapidly and effectively locate the position of an image block of a target scene, by using the attribute information marked by the OpenStreetMap data. Finally, we cropped the original remote sensing images based on the location information and obtained most of the images of the CLRS.

**Figure 4:**
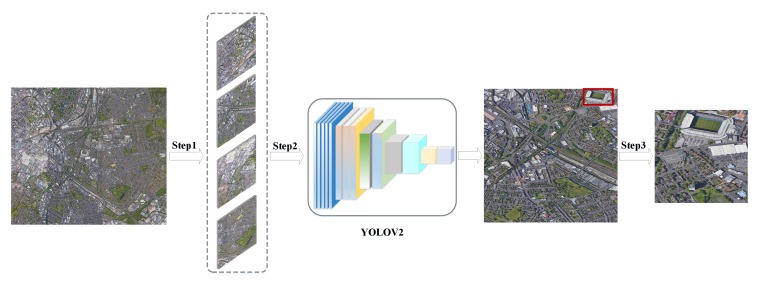
CLRS construction process based on the target detection pretrained model. Step1: Cropping the remote sensing image into 1024×1024 to satisfy the model input size. Step2: The target location information is obtained by YOLOV2 detection. Step3: The image was cropped to 256×256 size according to the detected coordinate range.

There are two major shortcomings in the method of constructing a remote sensing database based on the OpenStreetMap data. First, there is unavoidable error labeling information in the OpenStreetMap data, which needs to be manually screened. Second, some regions may not have labeled information, which makes it impossible to crop the images of these regions. To address the above issues, we have noticed that many excellent target detection pretrained models have been open sourced, including the YOLOV3 [26] model pretrained on the MS-COCO [27] dataset, the SSD [28] model pretrained on the PASCALVOC [29] dataset, etc. Through detection, the specific position of a target scene in a remote sensing image can be located and the boundary box can be drawn around the object so that the target of interest in the image can be extracted. However, there are only a few open-source pretrained detection models in the remote sensing community. The YOLOV2 [30] model, which has been trained on the DOTA [31] dataset is able to detect 15 scene categories: *plane, ship, storage tank, baseball diamond, tennis court, basketball court, ground track field, harbor, bridge, large vehicle, small vehicle, helicopter, roundabout, soccer ball field and swimming pool*. Therefore, in this study, we use the trained YOLOV2 model to locate the specific position of the target scene in the original remote sensing image (as shown in Figure 4). This method can quickly and effectively help us obtain the images of the CLRS. It is worth noting that there are two situations in the position range of the detected object: at the center of the remote sensing image or at the boundary of the remote sensing image. Therefore, there are two principles for cropping images.

**Figure 5:**
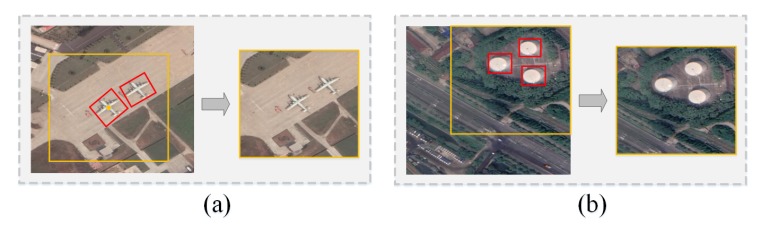
Cropping principles. (**a**) Detected object is at the center of the remote sensing image: crop the image according to the center of the object. (**b**) Detected object is on the edge of the remote sensing image: crop the image according to the boundary of remote sensing image.

(1) As shown in Figure 5a, if the detected object is at the center of the remote sensing image, the 256×256 image is cropped with the center of the detected object boundary box as the starting point. If other similar objects are included in the boundary box, only one image can be output.
(2) If the detected object is on the edge of the remote sensing image (as shown in Figure 5b), and cropping cannot be conducted with the object as the center, then the image is cropped according to the boundary of the remote sensing image, as long as the object is included in the 256×256 area.

## 3. The Proposed CLRS Dataset

**Figure 6:**
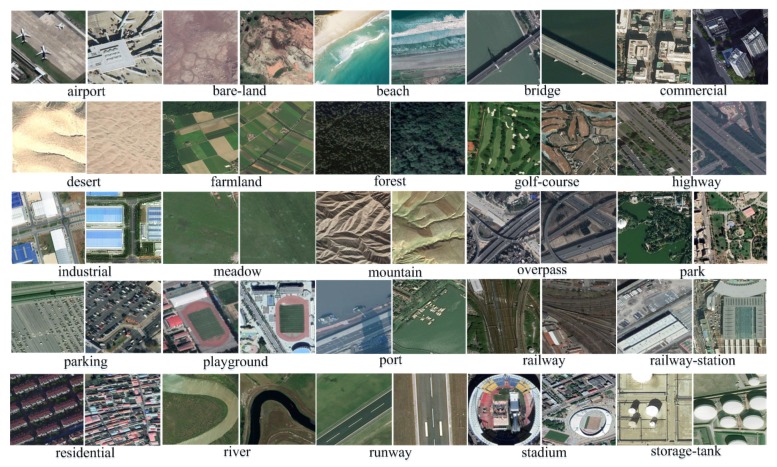
Some example images from CLRS data set: There are 15000 images within 25 classes. For each scene class, there are 600 samples. Two examples of per class are shown.

The proposed CLRS dataset consists of 15,000 remote sensing images divided into 25 scene classes, namely, *airport, bare-land, beach, bridge, commercial, desert, farmland, forest, golf-course, highway, industrial, meadow, mountain, overpass, park, parking, playground, port, railway, railway-station, residential, river, runway, stadium, and storage-tank*. Figure 6 shows samples of each class. Each class has 600 images, and the image size is 256×256. The resolution of the images ranges from 0.26 m to 8.85 m. The 15,000 images were collected from more than 100 countries and regions around the world.

Compared with the existing datasets, the CLRS has the following characteristics.

**Figure 7:**
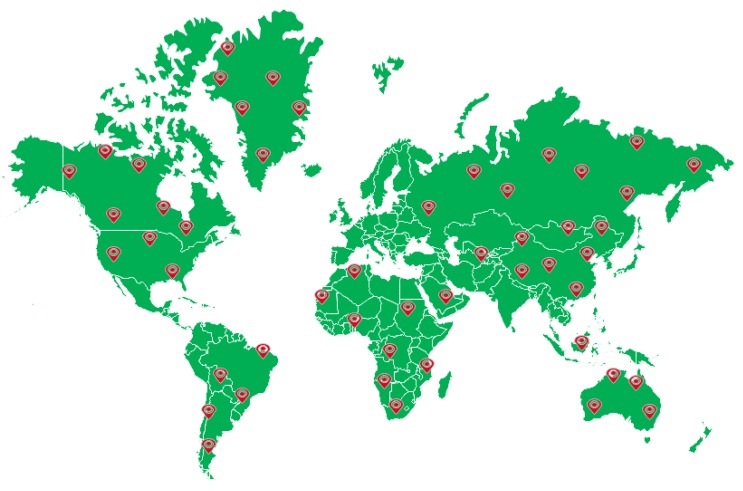
CLRS image acquisition area map; red marker points indicate images collected from the area.

**Figure 8:**
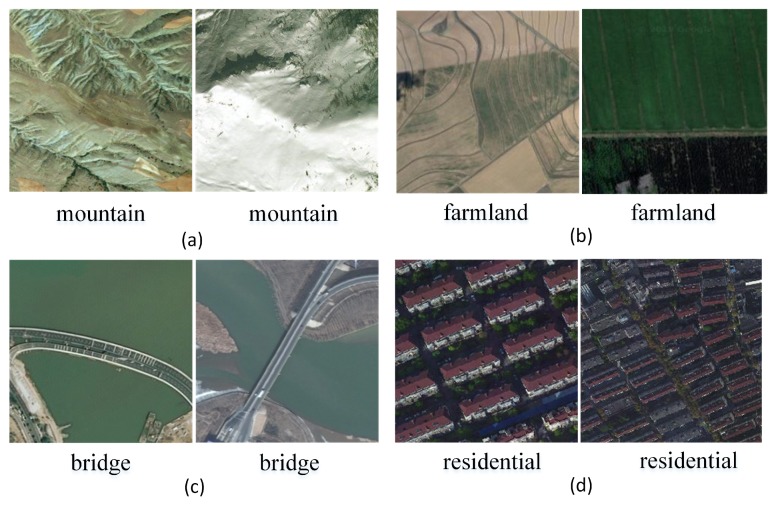
Higher intraclass diversity. (**a**) Instances of the same category in different seasons. (**b**) Instances of the same category in different climates and geographical environment. (**c**) Instances of the same category in different cultures and architectural styles. (**d**) Instances of the same category in different resolutions.

**Figure 9:**
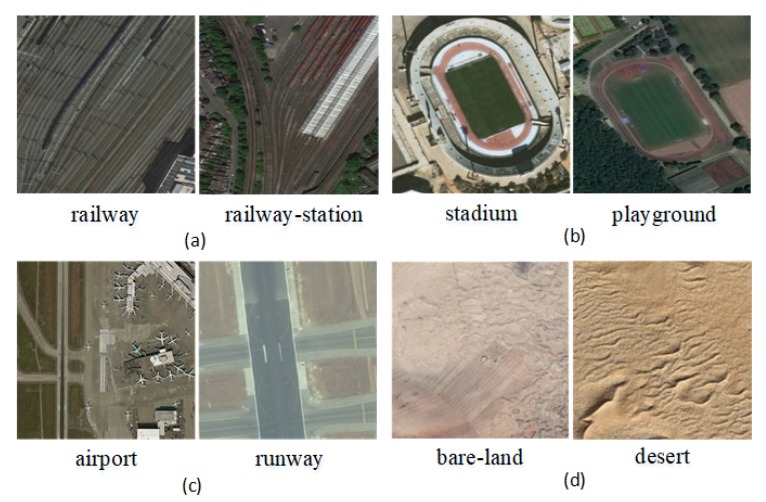
Larger interclass similarity. (**a**) Similar structures between different categories. (**b**) Similar objects between different categories. (**c**) Similar background between different categories. (**d**) Similar textures between different categories.

(1) *Multisource*. To meet the requirements of the deep learning model regarding the diversity of the samples in the dataset, the CLRS ensures the diversity and representativeness of the samples in the collection. In the same way as most of the existing datasets images are collected, such as AID++ [23], RSD46-WHU [32,33], etc., CLRS images are mainly collected from Google Earth, Bing Map, Google Map, and Tianditu, which use different remote imaging sensors. Therefore, the CLRS images are multisource and provide rich sample data.
(2) *The samples within the class are more diverse*. During the acquisition process, the image is considered for various factors, such as the illumination, background, time, scale, and angle. The image locations are widely distributed worldwide, including major cities and regions in Asia, Africa, Europe, North America, South America, and Oceania (as shown in Figure 7). These factors remarkably increase the intraclass diversity of the CLRS samples (see Figure 8). Figure 8a shows the differences in the same category due to seasonal changes. In Figure 8b, the effects of the climate and geographical environment can also lead to large variations in the same category of objects. In Figure 8c, we show sample differences due to the different cultures and architectural styles of different countries. In Figure 8d, we display two samples of the same scene category with different resolutions.
(3) *The difference between classes is smaller*. Given that the scenes in actual applications are often similar, the CLRS also selects some similar scene categories (as shown in Figure 9) to narrow the interclass differences of the CLRS. The main difference in Figure 9a is that the railway station not only has the railway but also the station. In Figure 9b, the stadium has the surrounding buildings except for the playground. The airport not only has many planes but also has a runway, as shown in Figure 9c. In Figure 9d, the bare land has some artificial traces, but the desert does not. The CLRS has higher interclass similarity and is closer to the actual remote sensing image scene classification task.
(4) *The CLRS provides the training batch partitioning standard*. The existing datasets lack training batch partitioning standards, and thus they cannot be used to evaluate and compare the performance of different continual learning algorithms in remote sensing image scene classification. The CLRS provides a set of training batch partitioning standards. Each CLRS image comprehensively records the spatial resolution of the image, and the resolution range of each type of image is counted. Based on the resolution, each type of image is divided into three levels. Each level has 200 images. Table 2 presents the resolution range of the three levels for each type of image. Each image will be named in the following format to facilitate the training batch division: *Category_Number_Resolution Level_Resolution Size.tif*.

Table 3 summarizes the comparison of several important factors between the CLRS and the existing scene classification databases. The CLRS has its advantages in data source, location distribution, and a training batch partitioning standard. In particular, the CLRS has a training batch partitioning standard, while other datasets are static benchmarks without a training batch partitioning standard. This is also the most important advantage of the CLRS as the benchmark of continual learning in remote sensing image scene classification. In terms of scene classes, total images, and spatial resolution, the NWPU-RESISC45 is larger than the CLRS. In future work, we will expand the number of scene classes and images of the CLRS.

## 4. Experiment

### 4.1. Training Batch Partitioning in Three Scenarios

The CLRS clearly gives the training batch partitioning and their corresponding labels in different scenarios. Therefore, researchers can test the related algorithms under the same standards. Specifically, the CLRS divides each image category into three levels according to the resolution. Based on these levels, the training batches can be divided into the following scenarios: the NI scenario, the NC scenario, and the NIC scenario. We randomly take 50 images from each of the three levels of each type of scene class as the test set, at a ratio of 3:1. The test set has 3750 images, including all 25 scene classes of the CLRS. The test set is fixed in the three scenarios. As the training batches increase for the model’s sequential learning, the model learns more and more classes or instances. However, the classification problem is unique, due to the fixed test set. After each batch is trained, the performance is evaluated on the whole test set, including the scene classes that have not yet been seen, which allows us to better compare the trends and characteristics of the model learning behavior [35]. Specifically, the training batch partitioning under the three scenarios is as follows.

- *NI scenario*: The remaining images can be divided into batch1, batch2, and batch3 for training. Each training batch has 25 scene classes and 3750 images. In the NI scenario, all the scene classes in the test set have appeared in the training set, that is, all the classes are known. The classifier only needs to classify 25 classes in each batch and does not need to distinguish new classes.
- *NC scenario*: Considering the smoothness between training batches, the NC scenario randomly divides the 25 scene classes into five copies, each of which contains all the images of the three levels in the five scene classes to simplify the difficulty. Each training batch contains five scene classes and 2250 images. In the NC scenario, the test set contains scene classes that have not yet appeared in the training set. A classifier needs to learn to distinguish not only all the classes it has seen so far but also those that have never appeared before. Therefore, the NC scenario is more difficult than the NI scenario.
- *NIC scenario*: Considering the five training batches of the NC scenario and the three training batches of the NI scenario, the NIC scenario can be divided into 15 batches. Each training batch has five scene classes and 750 images. In this scenario, the training batch sequence is longer (15 batches), and the model will continue to consolidate the previously learned knowledge while continuously learning new scene classes. Among the scenarios, the NIC scenario is the closest to real world remote sensing image scene classification and the most difficult of the three scenarios.

### 4.2. Baseline Methods

We test several mainstream continual learning methods on the CLRS, including Elastic Weights Consolidation (EWC) [14], Learning Without Forgetting (LWF) [16], and CopyWeights with Re-init (CWR) [22], and classic methods, including the Naive approach. EWC is a regularized continual learning method. It measures the important parameters of old batches using a Fisher information matrix, and in the process of training new batches, the updating of important parameters of old batches will be punished to reduce catastrophic forgetting of the old batches. LWF can be regarded as a combination of knowledge distillation [36] and Fine-tuning [37]. Based on the idea of knowledge distillation, the output of the model on the old batches is recorded as expert knowledge, to guide the learning of the new batches. The stability of the output of the old batches is constantly enhanced while learning the new batches, thus ensuring the performance of the model on the old batches and reducing forgetting. CWR is a structured continual learning strategy, which can share the parameters before the output layer. When the model learns a new batch, the neuron structure of the output layer of the old batches will be frozen, and the model will automatically expand the new neuron structure to learn new categories so that it can avoid forgetting during long batch sequences. The Naive approach continues using Stochastic Gradient Descent (SGD) training when new batches are available, which is also the most straightforward way of continual learning. However, the parameter space of the old batches will be overwritten after learning the new batches, as there is no mechanism to control forgetting. Therefore, the Naive approach can be used as a baseline to compare the control of forgetting with the continual learning methods. Finally, we compared the performance of multitask joint training, namely, we used Cumulative training [38] (the current batch data are mixed with all previous ones for training) as the baseline for evaluating the difficulty of a sequential batch.

### 4.3. Evaluation Metrics

We utilize the classification accuracy to estimate the performance of different methods. In order to eliminate the effect of training batches order on the results, we only randomly disturb the training batch order five times to reduce the computational complexity in the NI scenario and the NC scenario. We only scramble the training batch order three times due to the longer training batch sequence of the NIC scenario. The final result of each method is the average of the total number of runs. As the model automatically expands the new neuron structure to learn new categories, CWR is only suitable for use in the NC and NIC scenarios [35] (There are no new categories in NI scenarios). Therefore, in the NI scenario, we do not report the test accuracy of the CWR method.

### 4.4. Parameter Settings

We use ResNet [39] with 19 layers as the classification network in the experiment. All methods share the same network structure for fair comparison. Data augmentation and dropout are used to prevent model overfitting. The dropout value is set to 0.5. All parameters are initialized with Xavier. We use Stochastic Gradient Descent (SGD) to optimize the network. The learning rate is set to 0.001 and the batchsize is set to 64. In the all compared methods, the EWC and the LWF have hyper parameter λ. For the EWC method, we find the optimal hyper parameter with a greedy search. The optimal value of the hyper parameter λ is 15 in the NI scenario and the NC scenario, and 5 in the NIC scenario. In the LWF method, Davide Maltoni and Vincenzo Lomonaco [35] proposed that the value of λ should increase proportionally as the sequence of batches increases. Therefore, we also determine the optimal hyper parameter of each training batch by search. The λ values for each training batch are set as follows. In the NI scenario, $\lambda_{1}=0$, $\lambda_{2}=\frac{2}{3}$, and $\lambda_{3}=\frac{3}{4}$. In the NC scenario, $\lambda_{1}=0$, $\lambda_{2}=\frac{2}{3}$, $\lambda_{3}=\frac{3}{4}$, $\lambda_{4}=\frac{4}{5}$, and $\lambda_{5}=\frac{5}{6}$. In the NIC scenario, $\lambda_{1}=0$, $\lambda_{2}=\frac{2}{3}$, $\lambda_{3}=\frac{3}{4}$, ⋯, and $\lambda_{15}=\frac{15}{16}$.

### 4.5. Experiment Results and Analysis

#### 4.5.1. The NI Scenario

**Figure 10:**
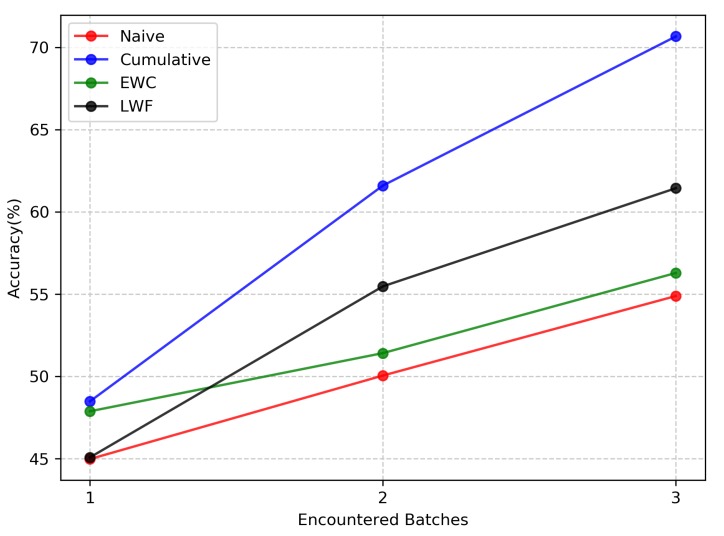
Test accuracy of the four methods on the New Instances (NI) scenario. The final result of each method is the average of disturbing the training batches order five times. EWC = Elastic Weights Consolidation; LWF = Learning Without Forgetting.

Figure 10 shows the test results of the four methods in the NI scenario. The accuracy curves of the four methods all show an upward trend. This demonstrates that with different instances of the same scene category appearing in the subsequent training batches, the four methods can continuously refine and consolidate the knowledge of the category that has been learned. The Cumulative approach, which mixes data from old and new batches together, achieves the ideal performance that a continual learning approach should achieve. After learning three training batches, LWF performs better than EWC and Naive method. The reason is that the three training batches distributions in the NI scenario are relatively similar, and the data of the new batch can effectively imitate the output of the model on the old batch through knowledge distillation. Therefore, LWF can well protect the performance of the model on the old batch. For EWC, the updates of the important parameters for the old batch are small when the new batch data distribution does not change much, thus protecting the performance on the old batch. However, in terms of accuracy, EWC is 5.15% lower than LWF after learning the third training batch. This further shows that LWF is better than EWC at protecting the performance of old batch. The Naive method is the worst performing of the three continual learning methods because it has no strategies to avoid forgetting. However, the forgetting is not obvious here. We also noticed that the performances of the EWC and Naive methods are very close. Although the parameter space of the Naive method is overwritten after a new batch is learned, the solution space of the three training batches is relatively close, and the model has a good initialization parameter when learning new batches. This model can make use of the features learned from the old batch to help improve the learning of the new batch, which is similar to using the pretrained model to fine-tune.

#### 4.5.2. The NC Scenario

**Figure 11:**
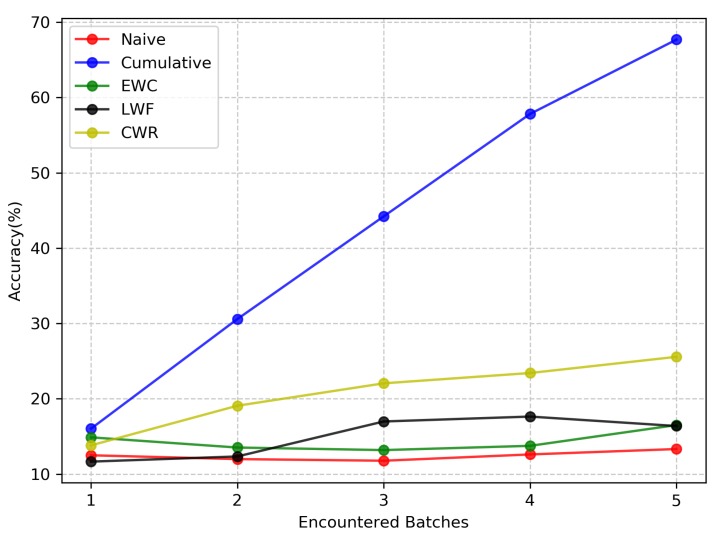
Test accuracy of the five methods on the New Classes (NC) scenario. The final result of each method is the average of scrambling the training batches order five times. CWR = CopyWeights with Re-init.

As shown in Figure 11, in terms of controlling forgetting, the test accuracies of the three continual learning methods EWC, LWF, and CWR in the NC scenario are also unsatisfactory. However, compared with the Naive method, the three continual learning methods are evidently superior to the Naive method. In the NC scenario, the accuracy of the Naive method is always approximately 12.44% due to the absence of any measures to control forgetting. This indicates that after the model has learned the new category, the parameter space of the old batch is completely covered by the parameter space of the new batch, which shows that the previous category is completely forgotten. After learning the fourth training batch, LWF still performs better than EWC in the NC scenario. However, on the fifth training batch, the performances of LWF and EWC are close. As the number of training batches increases, the difference between the distribution of the new batch and the old batch becomes larger, and the output on the old batch obtained through knowledge distillation will also become inaccurate, which will result in performance degradation of LWF. The same is true for EWC. It is difficult for EWC to find a common parameter subspace that satisfies all batches requirements. When using more capacity to remember previous training batches or learn current training batches, the parameter space between training batches becomes entangled. In the NC scenario, CWR is better than EWC and LWF on long sequence batches. It protects the performance of the old batch by freezing its neurons before learning a new batch. However, this approach also reduces the ability to flexibly learn new batches. In addition, we also noticed that after learning the five batches, CWR’s testing accuracy is only 25.57%, which is less than half of the Cumulative approach’s accuracy. Therefore, this also illustrates the difficulty of classifying continuous batches in complex scenarios.

#### 4.5.3. The NIC Scenario

**Figure 12:**
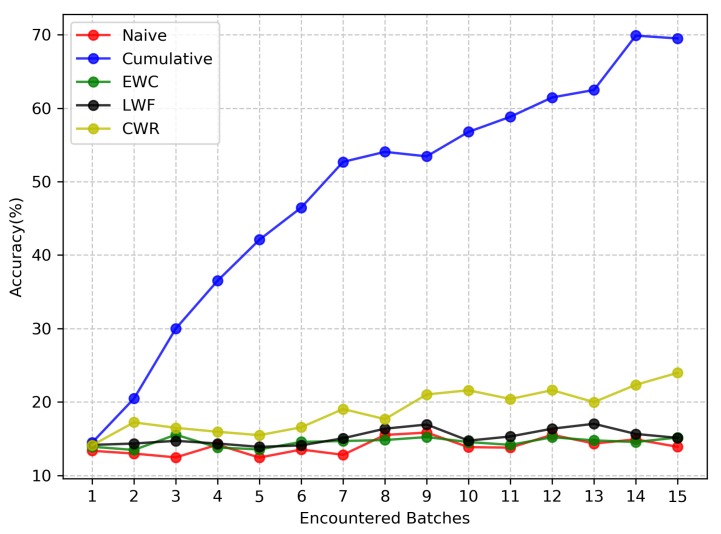
Test accuracy of the five methods on the New Instances and Classes (NIC) scenario. The final result of each method is the average of confusing the training batches order three times.

Figure 12 illustrates the average accuracy of the five methods in the NIC scenario. The Naive method cannot avoid forgetting when the model learns a longer sequence of batches. However, CWR is still very good at avoiding forgetting. In addition to the Cumulative approach, the performance of CWR is optimal among all continual learning methods. This demonstrates that is effective to freeze the neuron structure of the old batch to protect the performance of the model on the old batch and overcome the catastrophic forgetting of long sequence batches. Both EWC and LWF perform poorly in the NIC scenario. After learning batch8, the test accuracy of EWC is similar to that of the Naive method. This also shows that as the model continuously learns new batches, the shared parameter subspace of new batches and old batches is decreasing, and the cumulative error of the important parameters is increasing, which leads to the failure of EWC to protect the performance of longer sequence batches. The performance of LWF for a long sequence batch is better than EWC; however, it is still not satisfactory. Through knowledge distillation, the new batch data are used to imitate the output of models on old batches as much as possible. When the training batch sequence is longer and the training batch distribution is more diverse, the performance of LWF will decline. In addition, we can see that due to the complexity of the NIC scenario, the accuracy difference between the continual learning method and Cumulative approach is still quite large. Therefore, developing an emerging continual learning method to improve the test accuracy in this scenario is necessary.

### 4.6. Discussion

Based on the above experimental results, we can find that the test results on our dataset, the CLRS, can truly reflect the shortcomings of the existing continual learning methods. Specifically, the CWR method can perform well in both NC and NIC scenarios, which shows that freezing the neuron structure of old batches is a very effective way to overcome forgetting. However, this approach also comes at the expense of the flexibility of the network structure. The EWC method performs poorly on the longer sequences of batches. The reason is that as the number of training batches increases, the regularized strategy has difficulty finding a common solution space that meets all of the training batch requirements. The LWF method performs better than the EWC method on the longer sequence batches; however, the LWF method has the same disadvantages as the EWC method. As the distribution difference between the new batch and the old batch increases, the error between the output of the old batch obtained by knowledge distillation and the real output will also increase gradually, which will lead to poor performance. Although the performances of several continual learning methods are not satisfactory, we also note that the performances of three continual learning methods, i.e., the LWF method, the EWC method, and the CWR method, are still much improved over the Naive method that has no strategies to avoid forgetting. In addition, the performance of several continual learning methods is much worse than the Cumulative method (the ideal performance that continual learning methods should achieve). This also shows that the development of emerging continual learning methods with controlled forgetting strategies has important research value for improving the performance of remote sensing image scene classification.

## 5. Conclusions

In this paper, we first analyzed three application scenarios of continual learning in remote sensing, as well as gave the criteria for training batches partitioning in different scenarios to alleviate the current dilemma, where existing remote sensing datasets severely limit the development of continual learning in remote sensing image scene classification. Under this standard, we built a large-scale remote sensing image scene classification database called the CLRS. The purpose of the CLRS is to provide researchers with better data resources to develop state-of-the-art continual learning algorithms in remote sensing image scene classification. In addition, in order to reduce the costs of manual annotation, we proposed a new method of constructing a large-scale remote sensing image scene classification database based on the target detection pretrained model. Finally, we used the CLRS dataset to test and analyze several mainstream continual learning approaches in three continual learning scenarios, with results that can serve as a baseline for future works.

In future work, we will further expand the number of scene classes and images of the CLRS. At the same time, we will test new continual learning methods using the CLRS as the baseline for developing state-of-the-art algorithms in the field of remote sensing image scene classification. We will also extend the continual learning to other geo-spatial field such as graph convolutional networks on traffic predication [40] and geo big data analysis [41].

## Figures and Tables

**Table 1:** Remote sensing image scene category relation network. The left column is the parent class, and the right column is the subclasses contained by the parent class.

Parent Class	Subclasses
airport	airport, runway
highway	bridge, parking, parking_by_the_road, road, viaduct
port land	port
railway	railway, station
waters	beach, lake, river
unused land	bareland, desert, ice, rock, mountain
resident	mix, multi-family, single-family
arable land	dry land, paddy fields, terraces
grassland	meadow, shrub
woodland	forest
power station	solar, wind, hydraulic
factory	storage tank, works
mining area	mine, oilfield
commerce	commercial
religious land	church
sports land	baseball-field, basketball-field, golf-course, stadium, soccer field, tennis court
special land	cemetery
leisure land	amusement park, park, pool, square

**Table 2:** Three levels of resolution range for each type of image of CLRS.

Scene Categories	Level1 (m)	Level2 (m)	Level3 (m)
airport	0.26∼0.56	0.59∼0.98	0.98∼4.51
bare-land	0.39∼0.77	0.77∼1.11	1.11∼4.63
beach	0.34∼0.92	0.92∼1.70	1.71∼4.77
bridge	0.26∼0.77	0.77∼1.04	1.04∼4.88
commercial	0.44∼0.67	0.67∼0.95	0.95∼3.90
desert	0.44∼1.83	1.83∼4.28	4.28∼8.85
farmland	0.46∼1.62	1.62∼3.65	3.65∼8.17
forest	0.28∼0.46	0.46∼0.81	0.81∼4.76
golf-course	0.46∼0.92	0.92∼1.83	1.83∼8.17
highway	0.26∼0.67	0,67∼0.77	0.77∼3.23
industrial	0.46∼1.03	1.03∼1.86	1.86∼6.89
meadow	0.26∼0.73	0.73∼0.77	0.77∼2.27
mountain	0.26∼3.59	3.59∼3.81	3.81∼7.82
overpass	0.39∼0.81	0.81∼1.01	1.01∼3.82
park	0.43∼0.79	0.79∼1.03	1.03∼7.43
parking	0.22∼0.46	0.46∼0.73	0.73∼2.39
playground	0.26∼0.78	0.78∼1.02	1.02∼3.62
port	0.46∼0.73	0.73∼0.98	0.98∼8.17
railway	0.39∼0.73	0.73∼0.79	0.79∼3.89
railway station	0.39∼0.74	0.74∼0.92	0.92∼4.08
residential	0.45∼0.73	0.73∼1.15	1.16∼4.72
river	0.46∼1.78	1.78∼3.96	3.96∼8.17
runway	0.42∼0.49	0.49∼0.92	0.92∼2.39
stadium	0.28∼0.98	0.98∼1.90	1.91∼4.51
storage-tank	0.26∼0.71	0.71∼0.96	0.96∼4.51

**Table 3:** Comparison between the CLRS and the existing remote sensing image scene classification datasets.

Datasets	Scene Classes	Total Images	Spatial Resolution (m)	Data Source	Location Distribution	Training Batch Partitioning Standard
UCM [18]	21	2100	0.3	USGS	Urban areas in the United States	No
SIRI-WHU [34]	12	2400	2	Google Earth	mainly covers urban areas in China	No
AID [19]	30	10000	0.5∼8	Google Earth	mainly in China, the United States, England, etc.	No
NWPU-RESISC45 [20]	45	31500	0.2∼30	Google Earth	more than 100 countries	No
CLRS	25	15000	0.26∼8.85	Google Earth, Bing Map, Google Map, and Tianditu	more than 100 countries	Yes
